# The evil circle of poverty: a qualitative study of malaria and disability

**DOI:** 10.1186/1475-2875-11-15

**Published:** 2012-01-11

**Authors:** Benedicte Ingstad, Alister C Munthali, Stine H Braathen, Lisbet Grut

**Affiliations:** 1 Institute of Health and Society, Department of General Practice and Community Medicine, Section for Medical Anthropology, University of Oslo, P.O.Box 1130 Blindern, N-0318 Oslo, Norway; 2 Centre for Social Research, University of Malawi, P.O. Box 278, Zomba, Malawi; 3 SINTEF Technology and Society, PO Box 124, Blindern, N-0314 Oslo, Norway

**Keywords:** Health policy, Disability, Poverty, Rehabilitation

## Abstract

**Background:**

This article discusses the link between disability and malaria in a poor rural setting. Global malaria programmes and rehabilitation programmes are organized as vertical and separate programmes, and as such they focus on prevention, cure and control, and disability respectively. When looking at specific conditions and illnesses, the impairing long-term consequences of illness incidents during childhood are not questioned.

**Methods:**

The study design was ethnographic with an open, exploratory approach. Data were collected in Mangochi District in Malawi through qualitative in-depth interviews and participant observation.

**Results:**

Despite a local-based health service system, people living in poor rural areas are confronted with a multitude of barriers when accessing malaria prevention and treatment. Lack of skilled health personnel and equipment add to the general burden of poverty: insufficient knowledge about health care, problems connected to accessing the health facility in time, insufficient initiatives to prevent malaria attacks, and a general lack of attention to the long term disabling effects of a malaria attack.

**Conclusions:**

This study points to the importance of building malaria programmes, research and statistics that take into consideration the consequences of permanent impairment after a malaria attack, as well as the context of poverty in which they often occur. In order to do so, one needs to develop methods for detecting people whose disabilities are a direct result of not having received health services after a malaria episode. This may be done through qualitative approaches in local communities and should also be supplemented by suitable surveys in order to estimate the problem on a larger scale.

## Background

The importance of studying the relationship between malaria and disability emerged through findings from a comprehensive qualitative study on access to health care for people with disabilities in southern Africa. Malaria is one of the world's leading killers of children in southern Asia, tropical and sub-tropical Africa and parts of Latin America [1]. World Health Organization estimates that half of the world's population is at risk of malaria, which is an estimated 250 million cases every year [2]. While malaria in principle may strike everyone in affected areas, children who are poor, who have mothers with little or no education, and who live in the rural areas are more susceptible to contracting the disease and more likely to die from it [3]. Thus malaria, especially when it comes to fatal outcomes and disabilities, is to a large extent also a disease of poverty.

Although malaria for a long time has had its special vertical programmes in the WHO and the Ministries of Health of affected countries, [4] the "war against malaria" has not yet been won [5]. Malaria-specific programmes direct their focus on prevalence and incidence, prevention, treatment, mortality, and survival [6,7]. Disability due to cerebral malaria and the need for post-malaria neurological screening, especially in children under the age of five years, is only occasionally mentioned [8,9]. Nevertheless, visits to malaria stricken areas, especially rural and poor urban ones, reveal a number of people with disabilities who report that their impairment followed an episode of malaria. Estimates of percentage of children with permanent neurological damage as a result of cerebral malaria vary greatly, from 2% to 25% [10-13]. Reported after-effects of cerebral malaria in children are epilepsy, cerebral palsy, hearing impairment, visual impairment, intellectual impairment and attention deficit hyperactivity disorder (ADHD) [14]. Epilepsy as an initial complication often leads to physical and/or intellectual impairment when untreated.

Japanese encephalitis carries symptoms and consequences that in many ways resemble those of cerebral malaria and Lewthwaite et al [15] designed a questionnaire to assess the neurological and disabling impacts of Japanese encephalitis on children in resource-poor countries. Gladstone et al [16] tested a similar tool in Malawi, but without any mention of malaria as a cause of disability in the children who were screened for the purpose. In a study of epilepsy as an outcome of cerebral malaria in Malawi, Birbeck et al [17] found that 12 out of 132 children developed epilepsy while 28 of 121 cerebral malaria survivors developed additional neuro-disabilities. Other studies on malaria in Malawi do not mention disability as a possible outcome [18]. Nor do they discuss poverty as a contributing factor to non-compliance to prophylactic or therapeutic measures, but rather explain such behaviour as due to "women's weak knowledge" [19].

### Living condition and health facilities in Malawi

Malawi is among the countries in the world where malaria causes serious health problems. The whole population is at risk. In 2004, about 33% of all children in the district were estimated to have had malaria. Malawi's population is among the poorest in Africa. Over half the 15 million [20] population is food insecure and dependent on rain-fed smallholder agriculture. Those who live along the lake shore supplement their diet and income by fishing from small boats. While Malawi's National Statistical Office indicates that 39% are living below the poverty line [21], Palmer [22] claims that as much as 65% of the population are unable to meet their daily consumption needs.

This study was conducted in Mangochi District, which borders Lake Malawi, and which has a population of 803,602 people. Though densely populated, the settlement pattern is scattered. Most people live below the poverty line [23]. This is quite a paradox in a landscape that seems green and fertile, but for most of the families the patches of land are too small to feed the family, and some households have no land of their own. Women do most of the agricultural work. If a woman has to stay at home and take care of a disabled child, this reduces the agricultural output. The most common income option for men is occasional work on other people's fishing boats.

Mangochi District has 49 health facilities, 23 are owned and run by the government, the rest by NGOs and private actors. The lower level health facilities experience irregular and incomplete medicine supplies, they are understaffed and with high turnover among staff. The total number of health workers in the district was 1,453 in 2007, six of them being physicians. The current staffing levels at health facilities in Malawi are the lowest in the region and are threatened with collapse by a growing human resource crisis. Despite scarce resources, some improvements have taken place in recent years. Immunization programmes have eliminated neonatal tetanus and polio, and as of 2010 60% of all households owned an insecticide-treated net to prevent malaria [24]. However, owning a net does not say very much about its use: who in the household is using the net and for what purpose? Is the net repaired when it is torn? Can the family afford to buy a new one when the one they have is torn?

### Theoretical framework - the evil circle of disability and poverty

The theoretical perspective for this study is to understand poverty as structural violence which unfolds through social and cultural institutions, and which is avoidable [25]. Structural violence occurs when people are kept in poverty because they are hindered from meeting their basic needs, fulfilling their potential and accessing fundamental social institutions such as political power, education, and health care. In compliance with this perspective disability is understood as a socially constructed phenomenon [26]. The "evil circle of poverty" as discussed by Yeo and More [26] occurs when poverty generates disability, and disability generates and keeps people in poverty respectively.

During this fieldwork, a strong link between disability, poverty and malaria became apparent. Thus the link between malaria, delayed or lack of prevention and treatment, and disability became an important issue to investigate further in the study. In this article some of the aspects of this relationship will be discussed.

## Methods

The fieldwork for this qualitative study took place in March 2009. The study is built on an open ethnographic approach [27]. An ethnographic approach facilitates the opportunity to study people's lives defined broadly, to learn about their life situation, the values they strive for and the constraints under which they make their choices. The attention is directed to reflect knowledge and the systems of meaning which surround people's lives [28].

One of the strengths of ethnographic approach is to incorporate and illuminate findings and topics that may not have been explicitly considered at the onset of the study. Since Mangochi District borders Lake Malawi malaria was expected to be a problem. However, malaria as a cause to disability was initially not the main focus of the study. The apparently strong relationship between disability and malaria came up as one of the findings.

Data were collected by open in-depth interviews. Both individual and group interviews were conducted. Individual interviews were conducted in people's homes and in the villages. The informants were identified with the help of the village chiefs. The chiefs were visited on arrival and gave the research team permission to come back for interviews the following days. The chiefs identified key informants to approach and other informants were found through chain referral sampling (snowballing) [29] where the person who was interviewed identified the next informant. In some villages a number of people with disabilities gathered at the chief's house when the research team came back for interviews. This gave an opportunity to do group interviews in addition to the individual interviews. All interviews lasted one to two hours. In all 64 adults with disabilities and/or guardians of disabled children were interviewed. The informants were poor villagers who had a physical, sensory or intellectual impairment. A previous history of malaria was not a criterion for informant selection. The research team spent two to three days in each village, and observation of the village life and people's living condition was done during this stay. In addition to interviews with people with disabilities 21 health care providers were interviewed: two medical doctors, four rehabilitation personnel, three health surveillance assistance, four community health workers, four traditional birth attendants, and four traditional healers. The interviews followed a guide and were organized as a structured conversation between one researcher and one or more informants. Three of the four researchers do not speak the local language and these interviews were facilitated through a professional interpreter.

Ethical clearance was obtained from ethical research committees in Norway and Malawi. Information on the study's purpose was given to the informants and oral consent was obtained.

## Results

### Health services and poverty

Cerebral malaria or severe fever in late pregnancy or in early childhood was given as an explanation to the disability in 38 of the 64 individual interviews. All of the informants in this study had the option of attending modern health facilities. However, the settlement pattern in the area is scattered, and most people have a long way to travel to reach a health facility. Ways of getting to health facilities are by bicycle, which most people have to rent or borrow, or by foot to the main road to catch a bus. Lack of money to pay for transport is a serious barrier for poor people. The mother of a 13-year-old girl told that her daughter had developed normally up to the age of five when she got cerebral malaria. The family lives in a village one day's walk from a health facility. The mother had hesitated to seek help, thinking the daughter would recover, but as she got worse the mother carried her to the health clinic. When they arrived at the health clinic the daughter was critically ill. When the daughter recovered she had lost her hearing ability and could no longer walk properly.

The few health clinics that exist in the area are understaffed and underequipped. Lack of personnel and medication may leave the patient untreated or may delay the treatment for several days. A father of a mentally impaired son told that he had taken his child to the local clinic when the fever had lasted for three days. Because the clinic was out of medication they were referred to the district hospital. This delayed the onset of treatment by another two days. Upon recovery the son had epilepsy and was spastic in his right arm and leg. Even when medication is available, the additional costs may represent a significant barrier. Parents of children with epilepsy have to bring the children to the health clinic in order to collect the medication. The costs for frequent transport to the clinic to pick up the medication were too much for many of them and after a while most of them had stopped going to the health clinic for medication.

All of the informants were aware that malaria is carried by mosquitoes. They were aware of the need for protection, and of the efficiency of modern medicines in prevention and treatment. The informants told that a clinic would be their first choice when seeking help for malaria. However, belief in witchcraft as a cause of malaria was also prevalent among those understanding the efficiency of modern medicines. Informants who had chosen to go to the local traditional healer instead of the clinic gave various explanations for their choice. One reason was lack of money for transport to the clinic. Another reason was the understanding that high and moderate fevers respectively have different causes and therefore requires different treatment.

The cases mentioned above illustrate the close connection between cerebral malaria, neurological sequela, and disability. This is especially critical in poor rural populations where the distance to the nearest health facility is far, the means of transport poor and expensive, and the existing health facilities understaffed and underequipped. This finding was corroborated in interviews with medical doctors and other health facility staff. According to the health personnel interviewed, learning impairment, cerebral palsy and epilepsy were the most common after-effects of malaria, often combined with intellectual impairment. When the affected child recovers it is classified as a survivor in the health clinic's statistics. On subsequent visits to health facilities, new conditions may not be attributed to malaria in infancy.

### Mosquito bed nets and poverty

In Malawi bed nets treated with insecticide are given free to children under five and to pregnant women. The nets are distributed through health facilities to pregnant women that come for antenatal care (ANC) and to children that are brought for infant check-up. Many expectant mothers choose to give birth at home as traditional birth attendants are active in the local communities. The traditional birth attendants lost their official authorization in 2009 but they are still used because they are cheap and accessible in the community. They represent a risk for delay in cases of maternal malaria attacks. Coverage for BCG vaccination is however high in the area (95%) which indicates that children that are born at home are brought to the health facility for infant check-up.

Once the net is in the house the question arises about who is going to use it. A single mother with three small children had chosen to use the bed net herself and let the children sleep without one. Her decision was made out of consideration for the whole family. She was the provider of her family. If one of her children got sick she could take care of the child and if necessary bring the child to the clinic. If she herself got sick her children would have no one to care for them. Fishermen using the bed nets for fishing were also observed during the stay in villages near the lake. A similar explanation was given by them: what use is it to avoid malaria if the family is starving? From the perspective of a household provider who strives to maintain a minimum standard of living, this choice is understandable.

Malaria protection is dependent on when and how the nets are being used. They are to be used at night during sleep and hung from the ceiling above the bed and tucked under the mattress to keep the net tight and the mosquitoes out. But the mosquitoes are most active in the evening when the villagers gather around the fire outside the huts for the evening meal. The poor families cannot afford mattresses, but sleep on the floor on mats woven of stiff reeds that easily cut through the nets. The climate is humid and the nets are easily torn, thus reducing the life span of the nets.

## Discussion

The role malaria seems to play as a disabling condition is insufficiently recorded in medical reports and statistics about malaria. A nationally representative study of malaria mortality in India [30] shows a considerable under-reporting of deaths from malaria. By ethnographic fieldwork it is possible to go behind the statistics and see how significant health issues such as compliance, attendance, cure and disability manifests themselves among the people concerned.

Based on the findings from this study, one may claim that the obstacle to the use of bed nets for the prevention of malaria in a poor area is first and foremost poverty. Fathers who use the children's bed nets for fishing, or mothers who decide to be the one to sleep under the net, do not do this out of lack of knowledge or bad attitudes. On the contrary, this can be understood as a rational choice rooted in poverty and structural barriers.

The families of the impaired malaria survivors expressed that they were resigned to their fate. They are victims of what Ribera and Hausmann-Muela [31] call cumulative vulnerability, a situation where one life challenge comes on top of many others and makes the family abandon hope of change. Even if they wanted to consult doctors for after-effects, they lacked the means for transport and the money to pay for treatment and follow-up. In addition, the clinics in resource poor areas are not sufficiently equipped, and lack qualified and stable health personnel, medication, and equipment. Yeo and More [26] interpret situations like these as being trapped by the evil circle of poverty, where poverty is a leading cause of disability and *vice versa*. Cumulative vulnerability fits well into this picture in that the interaction between many disadvantageous factors enforces this evil circle.

Through an ethnographic approach this study has brought forward people's stories about living with the disabling after effects of malaria. The core problem people face seems to be to reach health services in time and/or to comply with the prescribed use of bed nets. These barriers are to a large extent rooted in poverty and structural barriers, and less in lack of knowledge and bad attitudes, which has been a common approach in qualitative studies of malaria issues [32]. The disabled malaria survivors were victims of what is called the evil circle of disability and poverty [26] and of what Farmer [33] and others [34] call social suffering due to structural violence. A social suffering approach puts the blame for the suffering were it rightly belongs: on the social forces that generate poverty and force people to make choices that keep them from getting proper prevention and treatment for malaria as well as for disability.

Much of the development aid is organized along specialities in which sectors target one condition only. This works against linking disability and malaria issues. When programmes against malaria and for rehabilitation are designed as separate interventions, the planners and implementors of the different programmes do not join forces for joint action. The lives of people who are carrying these conditions, however, are not separated into compartments but consist of totalities where poverty, disability, health care, sorrow and happiness are closely linked together.

### Limitation

The data obtained in this study is not validated against medical records. There is a theoretical possibility that the conditions mentioned have not been malaria in all cases. This should be considered a possible limitation of this study. Nevertheless, malaria is highly prevalent in the area and people know the symptoms well. In addition, the concurrence between the information from health personnel and the information from the disabled informants supports the conclusion of this study.

## Conclusion

This study on disability and poverty has generated information on disabling effects of cerebral malaria as a consequence of poverty. The informants in this study are known to the health services as malaria survivors. However, they do not receive health services for the disabling after-effects because poverty in most cases has prevented them from seeking such help. This study should be supplemented with further ethnographic studies in order to analyze the underlying forces for people's coping strategies and the link between poverty, malaria and disability. It is also strongly recommended that large-scale representative surveys be carried out to determine the extent of the problem of disability following cerebral malaria. Such studies should focus on those who have initially been treated at a health facility and should also reach out to those who have not accessed health services. Similarly, it is recommended that actors responsible for programmes fighting malaria and actors responsible for rehabilitation programmes cooperate on how to improve the situation for disabled malaria survivors.

## Competing interests

The authors declare that they have no competing interests.

## Authors' contributions

BI, LG, and SHB are responsible for planning and study design. BI, LG, SHB, and AM participated in the data collection, the data analyses and the writing of the article. All authors read and approved the final manuscript.
